# Epidemiological profile and temporal trend of exogenous intoxications in children and adolescents

**DOI:** 10.1590/1984-0462/2022/40/2021004IN

**Published:** 2022-05-27

**Authors:** Mônica Thalia Brito de Melo, Gibson Barros de Almeida Santana, Matheus Henrique Almeida Rocha, Roberta Karolline de Souza Lima, Talles Alberto Bispo da Silva, Carlos Dornels Freire de Souza, Amanda Karine Barros Ferreira Rodrigues

**Affiliations:** a Universidade Federal de Alagoas, Department of Medicine, Arapiraca, AL, Brazil.

**Keywords:** Poisoning, Child, Adolescent, Intoxicação, Criança, Adolescente

## Abstract

**Objective:**

To describe the epidemiological profile and to analyze the trend in the incidence rate of exogenous poisoning concerning children and adolescents (0–19 years old) in the city of Arapiraca, Alagoas, Brazil, in the period from 2007 to 2015.

**Methods:**

Observational study with data extracted from the Notifiable Diseases Information System. The variables sex, age group, toxic agent, and circumstance were analyzed using descriptive statistics. For temporal analysis, cut-off rates of incidence/10,000 inhabitants were calculated and the inflection point regression model was used for analysis.

**Results:**

There were 5,539 cases of exogenous intoxication in individuals aged 0–19 years in the city, of which 53.1% (n=2,944) occurred in girls and 61.5% (n=3,405) in children aged 0–9 years. Medicines consisted in the main agent responsible for intoxications (28.5%; n=1,580), mainly by accidental use (18.2%; n=1,010). There was a significant increase in the events during the study period (Average Annual Percent Change: 12.7; 95%CI 1.1–25.6; p<0.001), with rates increasing from 56.52/10,000 inhabitants in 2007 to 56.64/10,000 inhabitants in 2015. The incidence of cases in girls increased from 57.34/10,000 inhabitants in 2007 to 62.27/10,000 inhabitants in 2015. In boys, the incidence of cases was stationary: 55.69/10,000 inhabitants to 50.9 /10,000 inhabitants in the same period.

**Conclusions:**

The study showed a higher frequency of cases in girls aged 0 to 4 years and an increasing trend in the incidence rate during the study period. Implementation of actions and strategies, with emphasis on health education, is needed in order to prevent cases of exogenous intoxication.

## Resumo

**Objetivo:**

Descrever o perfil epidemiológico e analisar a tendência da taxa de incidência por intoxicações exógenas ocorridas em crianças e adolescentes (0–19 anos) em Arapiraca, Alagoas, Brasil, no período de 2007 a 2015.

**Métodos:**

Realizou-se um estudo observacional com dados extraídos do Sistema de Informações de Agravos de Notificação. No componente descritivo, foram analisadas as variáveis sexo, faixa etária, agente tóxico e circunstância mediante estatística descritiva (frequência). Para a análise temporal, foram calculadas as taxas de incidência/10 mil habitantes e foi utilizado o modelo de regressão por pontos de inflexão para a análise.

**Resultados:**

Ocorreram 5.539 casos de intoxicações exógenas em indivíduos de 0 a 19 anos em Arapiraca (AL), dos quais 53,1% (n=2.944) eram do sexo feminino e 61,5% (n=3.405) eram crianças de 0 a 9 anos. Os principais agentes responsáveis pelas intoxicações foram medicamentos (28,5%; n=1.580) e a circunstância frequente foi uso acidental (18,2%; n=1.010). Houve variação do coeficiente de incidência geral, o qual apresentou comportamento temporal crescente (average annual percent change: 12,7; intervalo de confiança — IC95% 1,1–25,6; p<0,001) e cuja taxa passou de 56,52/10.000 habitantes para 56,64/10.000 habitantes. A incidência de casos, no sexo feminino, passou de 57,34/10.000 habitantes para 62,27/10.000 habitantes e apresentou tendência crescente. Já no sexo masculino, a incidência de casos passou de 55,69/10.000 habitantes para 50,99/10.000 habitantes e apresentou comportamento temporal estacionário.

**Conclusões:**

O estudo demonstrou maior frequência de casos no sexo feminino, na faixa de 0 a 4 anos, e tendência crescente da taxa de incidência. Recomenda-se a implementação de ações e estratégias, com destaque para a educação em saúde, a fim de prevenir casos de intoxicação exógena.

## INTRODUCTION

Exogenous intoxication can be defined as a pathological process stimulated by substances that cause an imbalance in body homeostasis and which is mediated by biochemical reactions.^1^ The clinical and/or biochemical consequences of intoxication are related to the exposure time and the concentration of the substance to which the individual was exposed.^2^ Commonly listed substances are drugs, medicines, food substances, plants, and household, agricultural, chemical, and industrial products.^3^

In children, exogenous intoxication is a frequent cause of admission to emergency care services worldwide.^4^ In Brazil, in 2015, there were 38,600 cases (5.97/10,000 inhabitants) of poisoning in the age group from 0 to 19 years, which represents 42.3% of the total intoxication cases reported in that year.^5^ Preschoolers are the most exposed group, as they spend a considerable part of the day at home, exposed to risks related to access to toxic substances.^6^ Conversely, intoxication in individuals aged 10 years or older is usually related to suicide attempts.^4^

In the Northeast, in 2015, there were 9,188 exogenous poisonings in individuals aged up to 19 years, representing an incidence of 4.67/10,000 inhabitants.^5,7^ The distribution of exogenous poisonings in children and adolescents occurs heterogeneously and is concentrated in the states of Pernambuco (11.21/10,000 inhabitants; 3,546 cases) and Alagoas (9.10/10,000 inhabitants; 1,145 cases).^5,7^ The states of Maranhão and Sergipe have the lowest rates (0.72/10,000 inhabitants; 201 cases; and 1.79/10,000 inhabitants; 141 cases, respectively).^5,7^ Arapiraca, the study location of the present article, is the second most important and populous municipality in the state of Alagoas. It serves the entire 2^nd^ health macro-region, which comprises more than 46 municipalities, with a population of over 1 million inhabitants.^8^ In addition, there are no studies on the topic in this city. 

Considering the vulnerability of children and adolescents to poisoning and exposure to different potentially toxic substances, this study aimed to describe the epidemiological profile and to analyze the trend in the incidence rate of exogenous poisoning concerning children and adolescents in Arapiraca, Alagoas, Brazil, in the period from 2007 to 2015.

## METHOD

This is an observational time-series study that analyzed exogenous poisonings in children and adolescents (aged 0-19 years) in the municipality of Arapiraca, Alagoas, during the 2007-2015 period.

The municipality of Arapiraca is located in the state of Alagoas, Northeast region of Brazil. It occupies an area of 345.655km^2^, which makes it the second largest city in Alagoas. It has a population of 219 thousand inhabitants (population density of 600.83 inhabitants/km^2^), of which over 70 thousand are in the age group of 0 to 19 years.^7^ Among those who are 10 years of age or older, approximately 110,000 have no education or have some elementary education. Moreover, the city stands out as the head office of the 2^nd^ health macro-region of Alagoas, which encompasses 46 municipalities, corresponding to a population of over 1 million inhabitants.^8^ Its social vulnerability index in 2010 was 0.371 and the municipal human development index was 0.649.^8,9^

Cases of exogenous intoxication were analyzed according to the toxic agent (medicines, pesticide, rodenticide, chemical, drugs of abuse, poisonous plant, food and drink, other and ignored) and circumstance (frequent, accidental and environmental use, therapeutic use, medical prescription, administration error, self-medication, abuse, food intake, suicide attempt, attempted abortion, violence/homicide, other and ignored), according to sex (boys and girls) and age group (0 to 19 years).

In addition, to estimate the incidence rate of poisonings, Equation 1 was used as follows:

(1) $$\text{Annual incidence rate}=\frac{\text{Number of cases in target population (0-19 year old) in location ad year}}{\text{Population 0-19 living in the location and year}}\times 10,000$$

**Chart 1:**
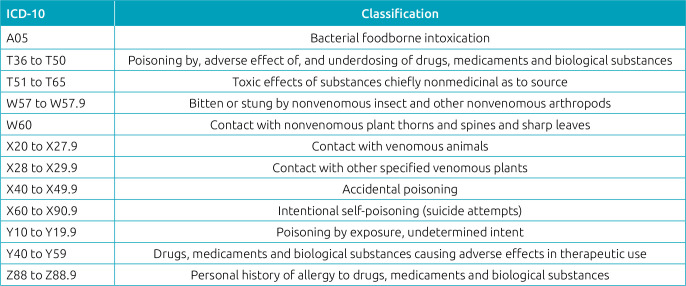
International statistical classification of diseases and health-related problems according to exogenous intoxication.

The records of the cases were extracted from the Notifiable Diseases Information System (*Sistema de Informação de Agravos de Notificação* – SINAN) of the Brazilian Health Informatics Department of the Ministry of Health (DATASUS).^7^ The International Classification of Diseases (ICD 10) was considered (Chart 1).^10^ The population data used in the study were collected from the Brazilian Institute of Geography and Statistics (IBGE),^8^ 2010 census, and from inter-census projections for other years.

ICD-10: International Statistical Classification of Diseases and Related Health Problems, 10th Revision

The analysis was carried out in two steps. The first consisted of a descriptive analysis, using absolute and relative frequency, with the variables “toxic agents” and “circumstance” to characterize the clinical/epidemiological profile. In the second step, the time-series analysis was performed using the inflection point regression model, which analyzes whether a line with multiple segments is ideal to demonstrate the periodic behavior of the data when compared with a straight line or with a lower number of segmentations. The Joinpoint Regression Program software (version 4.5.0.1, National Cancer Institute, Bethesda, Maryland, USA) was used. The trend was classified as stationary, increasing or decreasing, according to the slope of the regression line and statistical significance (p-value). The annual percent change (APC) and the average annual percent change (AAPC) were calculated, considering 95% confidence interval (95%CI) and 5% significance level. It should be noted that when no inflection points are formed, APC and AAPC values are the same. Thus, AAPC values only vary in the presence of inflections.

The present study used data from the public domain, which do not have identification of the subjects. Therefore, no approval by the Research Ethics Committee was necessary.

## RESULTS

In Arapiraca (state of Alagoas), during the study period, there were 5,539 cases of exogenous poisoning in individuals aged 0 to 19 years. Of these cases, 53.1% (n=2,944) were girls, and children aged zero to four years represented the largest group (43.7%; n=2,419), whereas adolescents aged 10 to 14 years accounted for a lower percentage (15.3%; n=847). The main toxic agent was medicine (28.5%; n=1,580) when disregarding the number of the ignored fields (37.7%; n=2,090). As for the circumstances of the poisonings, 18.2% (n=1,010) were accidental (Table 1).

**Table 1.** Epidemiological characterization of exogenous poisonings per sex according to age group, toxic agent, and circumstance in the municipality of Arapiraca, Alagoas, Brazil, from 2007 to 2015.

	Sex				Total	
	Boys		Girls			
	n	%	n	%	n	%
Age group (years)						
0 to 4	1,293	49.8	1,126	38.2	2,419	43.7
5 to 9	515	19.9	471	16.0	986	17.8
10 to 14	368	14.2	479	16.3	847	15.3
15 to 19	419	16.1	868	29.5	1,287	23.2
Toxic agent						
Medicine	649	25.0	931	31.6	1,580	28.5
Pesticides	39	1.5	41	1.4	80	1.4
Rodenticide	28	1.1	55	1.9	83	1.5
Chemicals	259	10.0	258	8.8	517	9.4
Drugs of abuse	31	1.2	29	1.0	60	1.1
Poisonous plant	62	2.4	50	1.7	112	2.0
Food and beverages	411	15.8	416	14.1	827	14.9
Other	101	3.9	89	3.0	190	3.5
Ignored	1,015	39.1	1,075	36.5	2,090	37.7
Circumstance						
Frequent use	19	0.7	16	0.54	35	0.6
Accidental	545	21.0	465	15.79	1,010	18.2
Environmental	62	2.4	49	1.66	111	2.0
Therapeutic use	278	10.7	224	7.61	502	9.0
Medical prescription	1	0.1	–	–	1	0.1
Administration error	21	0.8	24	0.82	45	0.8
Self-medication	25	1.0	41	1.39	66	1.2
Abuse	19	0.7	19	0.65	38	0.7
Food intake	392	15.1	409	13.89	801	14.5
Suicide attempt	104	4.0	512	17.39	616	11.1
Attempted abortion	–	–	3	0.10	3	0.0
Violence/homicide	2	0.1	2	0.07	4	0.1
Other	6	0.2	4	0.14	10	0.2
Ignored	1,121	43.2	1,176	39.95	2,297	41.5

Analysis of the quality of variables (ignored fields)						
	2007	2015	Period	APC	95%CI	p-value
Toxic agent	157	181	2007–2015	20.80	4.00–40.30	<0.001
Circumstance	269	181	2007–2015	14.80	-2.40–34.90	0.100

APC: annual percent change; 95%CI: 95% confidence interval.

Considering the toxic agent responsible for poisonings and the age group, children aged zero to four years were more intoxicated by medicines (24.9%; n=603) as well as adolescents aged 10 to 19 years. Conversely, poisoning in children aged five to nine years occurred as a result of food and beverages (23.2%; n=229). As for the circumstance of poisonings, suicide attempt was prevalent in adolescents aged 10 to 14 years (13.9%; n=118) and in those aged from 15 to 19 years (38.7%; n=498). In children under 10 years of age, poisoning occurred accidentally (44.5%; n=858) or by food intake (34.8%; n=526) (Table 2).

**Table 2.** Representation of poisoning cases per age group (years) stratified according to toxic agent and circumstance, in the municipality of Arapiraca, Alagoas, 2007 to 2015.

	0–4 years		5–9 years		10–14 years		15–19 years		Total	
	n	%	n	%	n	%	n	%	n	%
Toxic agent										
Medicine	603	24.9	211	21.4	261	30.8	505	39.2	1,580	28.5
Pesticides	20	0.8	9	0.9	16	1.9	35	2.7	80	1.4
Rodenticide	21	0.9	5	0.5	13	1.5	44	3.4	83	1.5
Chemicals	340	14.1	41	4.2	48	5.7	88	6.8	517	9.3
Drugs of abuse	5	0.2	2	0.2	6	0.7	47	3.7	60	1.1
Poisonous plant	29	1.2	13	1.3	22	2.6	48	3.8	112	2.0
Food and beverages	318	13.1	229	23.2	127	15	153	11.9	827	15.0
Other	99	4.1	48	4.9	20	2.4	23	1.8	190	3.4
Ignored	984	40.7	428	43.4	334	39.4	344	26.7	2,090	37.8
Circumstance										
Frequent use	13	0.5	6	0.6	7	0.8	9	0.7	35	0.6
Accidental	707	29.2	151	15.3	90	10.7	62	4.8	1,010	18.2
Environmental	26	1.1	11	1.1	18	2.1	56	4.4	111	2
Therapeutic use	247	10.2	97	9.9	87	10.3	71	5.5	502	9.1
Administration error	15	0.6	11	1.1	12	1.4	7	0.5	45	0.8
Self-medication	11	0.5	10	1	24	2.8	21	1.6	66	1.2
Abuse	4	0.2	1	0.1	7	0.8	26	2	38	0.6
Food intake	308	12.7	218	22.1	120	14.2	155	12	801	14.5
Suicide attempt	–	–	–	–	118	13.9	498	38.7	616	11.1
Attempted abortion	–	–	–	–	–	–	3	0.3	3	0.1
Violence/homicide/Medical prescription	2	0.1	–	–	1	0.1	2	0.2	5	0.1
Other	2	0.1	3	0.3	3	0.4	2	0.2	10	0.2
Ignored	1,084	44.8	478	48.5	360	42.5	375	29.1	2,297	41.5

**Figure 1.**
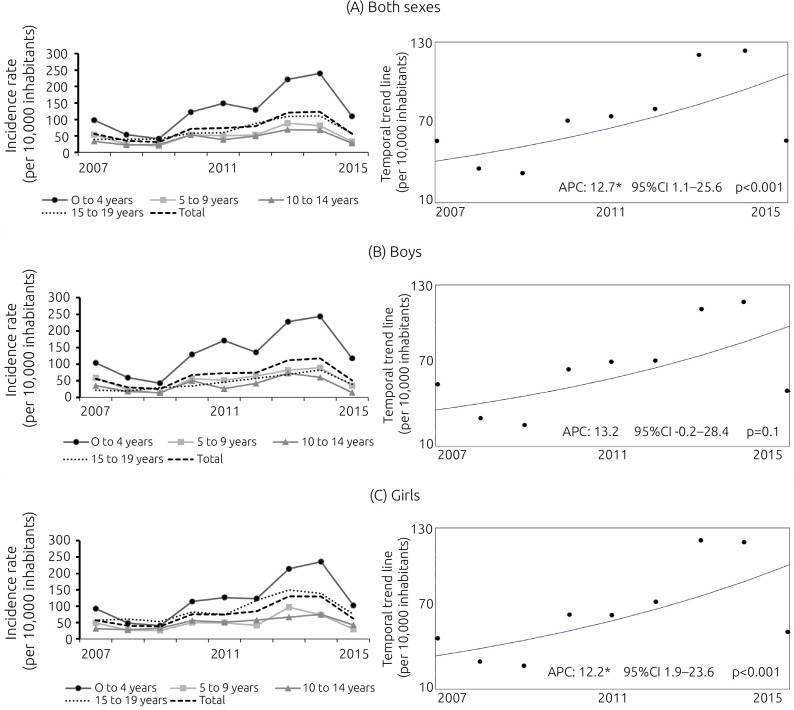
Incidence rate of exogenous poisoning by age group in the municipality of Arapiraca, Alagoas, Brazil, from 2007 to 2015.

Regarding the coefficient of incidence of exogenous poisoning, in the studied population, the average rate was 71.7 cases/10,000 inhabitants and there was variation during the analyzed period, in which an annual growth rate was observed from 31.49/10,000 inhabitants, in 2009, to 123.4/10,000 inhabitants in 2014, with a decrease to 56.64/10,000 inhabitants as of 2015. In boys aged zero to four years, the highest rate was observed in 2014: 244.09/10,000 inhabitants, followed by girls in the same age group and in same year: 235.45/10,000 inhabitants (Figure 1).

*Statistical significance (p<0.05); APC: annual percent change; 95%CI: 95% confidence interval.

When analyzing the total period, there was a higher incidence of cases in 2014 (123.4 cases/10,000 inhabitants) and an increasing trend in the incidence rate of exogenous poisoning in the studied population (AAPC: 12.7; 95%CI 1.1–25.6; p<0.001). The incidence of cases in girls ranged from 57.34/10,000 inhabitants to 62.27/10,000 inhabitants, with an increasing trend (APC: 12.2%; 95%CI 1.9–23.6; p<0.001). Conversely, as for boys, the incidence of cases ranged from 55.69/10,000 inhabitants to 50.9/10,000 inhabitants, presenting a stationary trend (APC: 13.2%; 95%CI -0.2–28.4; p<0.10). With regard to the analysis according to age group, the temporal modeling showed variation according to age. In the age group from zero to four years, the regression model showed an increasing temporal behavior (AAPC: 16.0; 95%CI 1.8–32.3; p<0.001), which is similar to the age group from 10 to 14 years (AAPC: 10.2; 95%CI: 0.1–21.3; p<0.001) (Table 3). 

**Table 3.** Analysis of incidence and trend of cases of hospitalizations for exogenous poisoning in children and adolescents according to sex and age group in the period 2007-2015 in Arapiraca, Alagoas, Brazil.

Descriptive analysis		Rates		Trend analysis				Trend
	n (%)	2007	2015	Period	APC	95%CI	p-value	
Sex								
Both	5,539	56.52	56.64	2007–2015	12.7%*	1.1–25.6	<0.001	Increasing
	(100)							
Boys	2,595	55.69	50.99	2007–2015	13.20%	-0.2–28.4	0.100	Stationary
	(46.8)							
Girls	2,944	57.34	62.27	2007–2015	12.2%*	1.9–23.6	<0.001	Increasing
	(53.2)							
Age group								
0 to 4	2,419	98.13	109.89	2007–2015	16.0%*	1.8–32.3	<0.001	Increasing
	(43.7)							
5 to 9	986	54.11	33	2007–2015	9.00%	-5.2–25.5	0.200	Stationary
	(17.8)							
10 to 14	847	33.49	28.4812	2007–2015	10.2%*	0.1–21.3	<0.001	Increasing
	(15.3)							
15 to 19	1,287	39.4	57.99	2007–2013	21.5%*	12.9–30.7	<0.001	Increasing
	(23.2)			2013–2015	-19.40%	-51.5–33.9	0.300	Stationary
				2007–2015	9.60%	0.6–20.9	0.100	Stationary

*Statistical significance (p<0.05); APC: annual percent change; 95%CI: 95% confidence interval.

## DISCUSSION

The study results demonstrated a predominance of exogenous poisoning in girls and in the age group from zero to four years. The main toxic agent was medicine and accidental use prevailed in all age groups when jointly analyzed. In the period from 2007 to 2015, 5,539 cases of exogenous poisoning were reported in Arapiraca (state of Alagoas), with an average of 615.44 per year and an instability in the incidence rates. In addition, there was a significant increasing trend in girls and in the age groups from zero to four years and from 10 to 14 years.

Worldwide, in 2017, there were over 2 million notifications of human exposure to toxic substances, among which more than 50% of acute intoxications were reported for children under 13 years of age.^11^ Although this study presents a higher incidence of poisoning in girls and in the age group from zero to four years, these data partially contrast with data from the national literature, according to which boys are more exposed to agents triggering intoxication.^6,12,13^ This can be understood from the cultural perspective, as society tends to allow families to educate boys with less surveillance, which results in greater number of accidents and deaths from external causes registered for this population.^14^

In studies carried out in Iran^15^ and India,^16^ the mean age of greater occurrence of exogenous poisonings was in children aged between zero and four years and in those aged between two and three years, respectively. Likewise, Brazilian studies conducted in the states of Belo Horizonte^6^ and Pernambuco^17^ demonstrated a higher occurrence of poisoning in children under five years of age. Due to cognitive immaturity, curiosity, and the development of motor skills, children are exposed to different situations according to their age group. For instance, the younger ones, when crawling, are subject to contact with chemicals on surfaces through their hands and knees, in such a way there can be oral and dermal absorption.^18^

Due to this context, there are regulations for the protection of children against toxic exposures in many countries such as specific contraindications to pharmaceutical products.^15^ However, data from the present study demonstrated that medicines were responsible for 28.53% of intoxications. There are probably different reasons, considering the easy access of children to medicines, their indiscriminate use, and the lack of studies on administered medications among children, which estimate their risk. In a study conducted in the city of Porto Alegre (state of Rio Grande do Sul, Brazil) on caregivers of children (n=50) who had been poisoned, it was found that the height of the toxic agent below 150 cm was 17 times more likely to be associated with a toxic event, and distraction on the part of the caregivers was 15 times more likely to be related to such occurrence.^19^ In the metropolitan region of Manaus (state of Amazonas, Brazil), in 2015, more than half of the households had hazardous products and a third of them stored such products without safety.^20^ For this reason, surveillance and protective measures, such as resistant packaging and safe storage of toxic substances, contribute to the prevention of accidental poisoning in childhood.

Another issue highlighted by the present findings is the high rate of attempted suicide, which is the main cause of exogenous intoxication among adolescents. This scenario is aggravated when estimates indicate that, for each attempt, there were between 20 and 30 previous attempts, and in only four of them care was sought. Thus, even official estimates do not represent reality, as they are underestimated.^21^ It has been shown that there is an association between an increased risk of suicide with the number of attempts as well as with shorter time intervals between them. Of the individuals admitted to the emergency room for suicide attempt, it is estimated that 30 to 60% are recurrences and 10 to 25% will do it again within a year.^22^

There are many risk factors related to suicide in adolescence: social isolation, intra-family violence, physical or sexual abuse, mental illness, stress, alcohol and drug use, lack of social support and health conditions, feelings of loneliness, previous suicide in the family, poverty, homosexuality, bullying, low self-esteem, and poor school performance, among others.^23^ Worldwide data show that most suicides occur among adolescents over 14 years of age, but in some countries there is an increase in those under 15 years of age.^24^

In the present study, ingestion of medications was the most used form of suicide attempt. These findings are in accordance with a study carried out in a hospital unit in the city of Ribeirão Preto (state of São Paulo, Brazil), in which 77.8% (n=56) of the cases were girls, predominantly between 15 and 19 years of age, and through ingestion of medicines.^25^ In a retrospective survey of 206 cases of suicide attempts due to drug intoxication, the classes that stood out the most were tranquilizers (25.5%), antidepressants (17%), and anticonvulsants (15%). As the use of drugs is considered a common method for suicide attempt, continuous monitoring is necessary, together with a careful assessment of patients’ mental and emotional state before prescribing such substances.^22^

The World Health Organization states that restricting access to the means of committing suicide, early identification and treatment of people with psychological disorders, and improving access to social and health services are effective strategies for prevention.^23^ In 2005, the Brazilian Ministry of Health formed a group responsible for formulating national policies on suicide prevention.^22^ A systematic review showed that the implementation of psychosocial interventions at school, in the community, and in health environments is an important preventive strategy against suicide attempts in young people.^26^

Furthermore, it is worth removing social stigmas toward the individual who attempts suicide. The search for a healthcare service after the attempt is influenced by the level of access and trust in the health system, the population’s stigma in relation to suicidal behavior, and the fear of criminalizing the act.^22^ Thus, it is important to use these tools to facilitate the access of those who attempt suicide to healthcare services as well as the development of prevention strategies.

It is worth highlighting that the age group from five to nine years suffered poisoning from contaminated food and beverages, a relevant circumstance regarding the causes of exogenous poisoning. This scenario can worsen in pandemic times, such as that caused by SARS-CoV-2, in which an increase in admission to poisoning centers since the beginning of the pandemic has been reported, related to exposure to cleaning products and disinfectants in an attempt to avoid virus contamination.^27^ The fear of becoming infected caused a dramatic change in the behavior of the population, with the inappropriate use of products such as chlorine for personal or food hygiene.^28^ Thus, individuals engage in high-risk practices related to erroneous food hygiene, which increases the frequency of adverse effects.

Comparing 2019 and 2020, the number of deaths from poisonings with methanol in Iran increased 11 times due to the dissemination of false information about alcohol consumption as a form of prevention against COVID-19.^28^ In addition, with the adoption of measures, such as the closing of schools and daycare centers by political authorities, children spend more time at home and their exposure to toxic substances, such as hydroalcoholic solutions, homemade products, and medicines, increases. Self-medication also stood out as a frequent event related to domestic accidents in the context of the pandemic, especially with regard to the use of drugs without scientific evidence.^29^ Thus, information on products that cause morbidity and mortality is an important element to guide health education initiatives.^17^

This study has limitations, among which the expanded approach to the problem is highlighted, including, in a single analysis, intentional and accidental cases of exogenous intoxication. Nevertheless, this approach is justified by the need to characterize exogenous intoxications in a broad sense, as there are no studies with the same focus in the city of Arapiraca. Other limitations were the use of secondary data and the high number of ignored/blank fields found in the variables “toxic agents” and “circumstances of intoxication.” In Brazil, there is an incidence of 4.8 million exogenous poisonings per year, of which 0.1 to 0.4% result in death. However, these cases are probably underestimated according to some indications.^30,31^ It is estimated that only 20% of cases of pesticide poisoning are actually registered, even though they consist in events of compulsory notification.^32^ It is noteworthy that this study identified a statistically significant growth trend in the ignored/blank fields in the period from 2007 to 2015.

Furthermore, healthcare services face difficulties in properly identifying intoxication as intentional and unintentional, in addition to the existence of underreporting for sociocultural and economic reasons and misclassification of suicide attempts.^31^ Moreover, the available epidemiological data regarding exogenous poisonings are scarce and present problems such as the lack of standardization in data collection and inadequate storage.^30^

The study showed a higher frequency in girls and a higher incidence in the age group from zero to four years throughout the analyzed period (2007–2015), with an increasing trend in the incidence of the event. Therefore, the implementation of actions and strategies is suggested, with emphasis on health education, to prevent cases of exogenous intoxication in addition to improving the completion of the notification form. This is because, with the poor quality of available data, formulating adequate prevention strategies in the municipality of Arapiraca becomes an unattainable goal, in such a way that measures for qualifying these pieces of information must be adopted.
